# Application of Artificial Intelligence in the Diagnosis, Treatment, and Prognostic Evaluation of Mediastinal Malignant Tumors

**DOI:** 10.3390/jcm12082818

**Published:** 2023-04-11

**Authors:** Jiyun Pang, Weigang Xiu, Xuelei Ma

**Affiliations:** 1 Division of Thoracic Tumor Multimodality Treatment, Cancer Center, West China Hospital, Sichuan University, Chengdu 610041, China; 2 State Key Laboratory of Biotherapy, Cancer Center, West China Hospital, Sichuan University, Chengdu 610041, China; 3 West China School of Medicine, Sichuan University, Chengdu 610041, China; 4 Department of Biotherapy, Cancer Center, West China Hospital, Sichuan University, Chengdu 610041, China

**Keywords:** artificial intelligence, mediastinal malignancy, deep learning, artificial neural network

## Abstract

Artificial intelligence (AI), also known as machine intelligence, is widely utilized in the medical field, promoting medical advances. Malignant tumors are the critical focus of medical research and improvement of clinical diagnosis and treatment. Mediastinal malignancy is an important tumor that attracts increasing attention today due to the difficulties in treatment. Combined with artificial intelligence, challenges from drug discovery to survival improvement are constantly being overcome. This article reviews the progress of the use of AI in the diagnosis, treatment, and prognostic prospects of mediastinal malignant tumors based on current literature findings.

## 1. Introduction

Artificial intelligence is a new intellectual ability that can simulate and expand human thinking and judgment [1]. As a comprehensive discipline developed through integrating computer science, psychology, linguistics, and other disciplines, artificial intelligence, alongside genetic engineering and nanoscience, is considered one of the three most state-of-the-art technologies in the world in the 21st century. Artificial intelligence was first proposed by the Dartmouth Institute in 1956 [2] and, in recent years, with advances in related technologies, it has reshaped every walk of life, encompassing numerous fields. The expansion of databases, the innovation of algorithms, and the improvement of hardware skills have laid a solid foundation for a wide range of applications of AI in the medical field.

**Figure 1:**
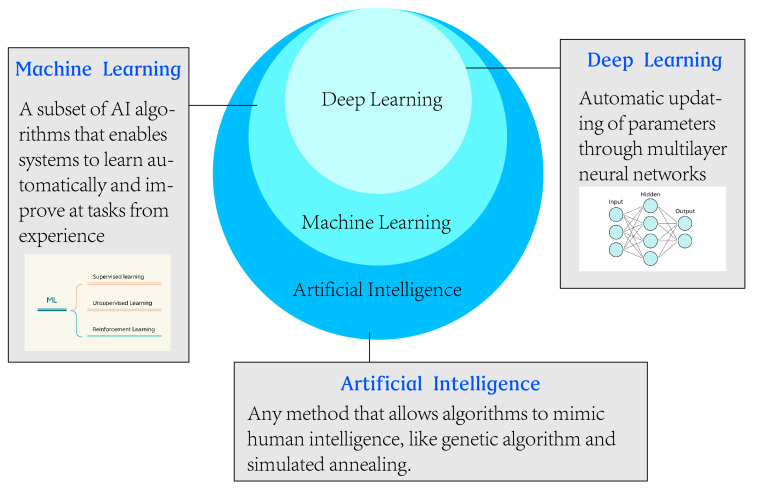
The differences and relationships among artificial intelligence (AI), machine learning (ML), and deep learning (DL).

Machine learning (ML) is a branch of artificial intelligence that uses structured training data to teach machines so that the machine can learn and adapt to create and modify its algorithms for new data to solve problems (Figure 1) [3]. Meanwhile, deep learning is a new field and a part of machine learning, and directly processes unstructured data, such as images and sounds, with representation learning based on artificial neural networks. It is rich with powerful computing, automation, and variability [4]. In recent years, deep learning algorithms have brought remarkable accomplishments, even exceeding human intelligence [5,6,7]. Among various deep learning algorithm models, the convolutional neural network (CNN) is the most popular deep learning architecture in medical imaging [8]. CNN has the potential of representation learning and effectively extracts, organizes, and classifies internal features of imaging data.

Cancer is the second most common disease specific to morbidity and mortality, and up to 19.2 million new malignant tumor cases are diagnosed every year [9], endangering human health and prompting to seek long-term remedies. The mediastinum consists of the left and right mediastinal pleura, organs, other structures, and connective tissue [10]. A mediastinal malignant tumor refers to a malignancy found in the mediastinal area, including neurogenic tumor, teratoma, thymoma, and germinoma [11]. Clinical manifestations of mediastinal malignant tumors are severe chest tightness and pain, different degrees of respiratory tract compression, and nervous system and cardiovascular symptoms depending on their locations [12,13]. Moreover, prompt diagnosis and treatment are of utmost importance, considering the complex origin and anatomical position. Using artificial intelligence can substantially improve the efficiency of preliminary diagnosis to prognostic analysis. However, artificial intelligence comes with challenges, such as data security and patient privacy. Furthermore, mediastinal malignancy is less studied compared to lung, breast, and other major cancers, and requires more attention and exploration [14]. To recapitulate, there are still prospects of applying AI in the diagnosis and treatment of mediastinal malignancy in the future with addressing challenges.

## 2. Diagnosing Mediastinal Malignant Tumors

### 2.1. Diagnosis Using Imaging

Before the advent of the first CT machine in the 1970s, mediastinal tumors were mostly diagnosed using chest radiography with a qualitative diagnosis rate of 30%. CT improved the diagnostic accuracy of localized tumors to >90%. Currently, ultrasonography, magnetic resonance imaging, thoracoscopy, and other imaging technologies have been used in screening, staging, and evaluating the efficacy of mediastinal malignant tumors. Computer-aided diagnosis (CAD) has also been adopted since its development in the 1980s [15]. However, these traditional image technologies also come with limitations. Furthermore, diagnosis using imaging technologies depends on the level of experience of diagnosticians, which is highly subjective, inevitably leading to a misdiagnosis at times. Furthermore, with the increase in population and disease screening awareness, radiologists need to process huge image data and deal with an increasing workload. Radiologists can only afford 3 to 4 s to read an image [16], and this time demand undoubtedly results in a high error rate and serious consequences in the long run. With the presently available automated and reproducible assessment capabilities through AI, challenges in diagnostic accuracy and doctors’ workload could be easily solved.

Two AI approaches can be adapted to the diagnostic imaging of malignant tumors [17]. The first method predefines tumor characteristics, such as tumor texture, volume, and shape based on mathematical equations, and then quantifies using computer programs [18]. The second method is deep learning, attracting greater attention in the medical field. Esteva et al. [19] classified skin lesions from clinical images using a deep convolutional neural network and found similar diagnostic accuracy to that of 21 qualified dermatologists. Deep learning is a highly reliable powerful tool to integrate with imaging technologies to reduce the workload of doctors.

The learning ability and reliability of AI have been proven, compared with the outcomes of various mediastinal malignant tumor diagnostic assays. Ozkan et al. [20] proposed a machine learning model for positron emission tomography with 2-deoxy-2- [fluorine-18] fluoro-D-glucose integrated with computed tomographic (18F-FDG PET/CT) images using a multilayer perception (MLP) classifier (ANN), which successfully predicted the low-risk and high-risk thymomas (AUC = 0.830) (Table S1). Dai et al. [21] associated computed tomography features with pathological tumor characteristics, and they found the random forest model highly efficient in diagnosing thymic carcinoma and high-risk thymoma, with a predictive accuracy of 94.73% using test data. Lin et al. [22] developed an AI model to predict the pathological subtypes of prevascular mediastinal tumors (PMTs). The model was able to identify lymphoma, thymoma, and thymic carcinoma with sensitivities of 52.9%, 74.2%, and 92.8%, respectively. These are some examples demonstrating that an ML algorithm combined with CT, X-ray, MRI, and other conventional imaging diagnostic methods could greatly improve detection efficiency and accuracy, and reduce the pressure on diagnosticians.

Furthermore, recent years have witnessed remarkable contributions of various novel imaging technologies utilizing artificial intelligence in the diagnosis of malignant tumors [17]. For instance, Chowdhary et al. [23] developed an AI-based prognostic model for patients with thymic epithelial tumors, achieving an overall accuracy of 84.2% and 87.0% in the training and validation cohorts, respectively, with an AUC of 0.90. Advanced imaging technologies such as multi-physical coupled imaging and contrast-enhanced ultrasound are being continuously explored and updated, demonstrating significant potential for development [24]. Combining these methods with advanced machine learning would advance further. Diagnostic imaging of mediastinal tumors has long been a challenge due to their diverse clinical behaviors, including the primary and metastatic nature of tumors, complex mediastinal structure locations, and occult onset. These new technologies with AI can capture the features of tissues, and can accurately identify subtle characteristics, such as microvascular invasion and lymph node metastasis. Through accurate diagnosis, personalized treatment can be easily designed [25].

AI can also be applied to laboratory parameters assessment. Some clinical cases may be misjudged using laboratory diagnostic assay outcomes; for example, due to false positive and false negative results. Incorporating AI in analyzing the laboratory indices, employing big data for one-to-one comparison would accurately identify patients with mediastinal tumor risk.

AI still has some deficiencies with the application of diagnostic imaging. For example, the black-box nature of AI makes the outcome partially interpretable and makes it difficult to quantify the prediction [26]. Detection results may also be related to input data, image quality, and other factors, and errors may still exist. AI can hopefully be further developed to escort the diagnostic imaging of mediastinal tumors.

### 2.2. Diagnosis by Pathological Examinations

Despite the advent of non-invasive techniques such as liquid biopsy, traditional histopathology remains the gold standard for the diagnosis of mediastinal malignancies. Histopathological or cytopathological diagnosis confirms the presence of tumor cells in patient specimens, identifies biomarkers of cancers and describes the tumor characteristics, such as types, stages, and grades [27]. Compared to imaging and other diagnostic methods, histopathological/cytopathological assessment clearly describes the characteristics of tumors and is widely used in the qualitative evaluation and classification of mediastinal tumors before surgery and after surgery. However, diagnosis using biopsy samples requires extensive experience and knowledge of pathologists, but experts in this field are scarce, and do not guarantee diagnostic accuracy. On the other hand, histopathological evaluation from fixation to staining requires a series of complex processes, and there is no strict standardization for these processes. With the rapid development of AI in clinical image evaluation, natural language extraction, and other aspects, the use of AI for pathological diagnosis has become the focus of tumor diagnosis, which has been widely applied for differentiating benign tumors from malignant tumors, and the grading and typing of several tumors, including mediastinal tumors [28]. Kalra et al., reported that AI can aid pathologists in accurately diagnosing thymoma [29].

The first step in the use of AI in pathology was made in the early 1950s, when Mellors and Silver suggested the use of automated pre-screening machines (microfluorescence scanners) to evaluate Pap smears [30]. Bostrom et al., who represented the pioneering work of many scientists, used various experimental image analyzers to automatically scan the clinical images [31]. As of yet, AI is competent in pathological diagnosis, especially in glandular and tumor classifications [32,33].

The deep CNN model of artificial intelligence is particularly efficient in extracting primary features from high-resolution pathological specimen images to accurately diagnose [34]. Presently, a variety of ML algorithm models have been employed in the pathological diagnosis of thymic cancer, prostate cancer, colon cancer, breast cancer, and other tumors, with satisfactory accuracy [35,36,37,38,39,40]. Using raw input data from virtual images of lung cancer biopsy stained with H and E, Coudray et al. [41] trained a CNN model to accurately predict six different gene mutations by analyzing specific histological patterns associated with various molecular subtypes of lung adenocarcinoma. Nevertheless, research on mediastinal tumors is lacking, and the application of AI would help in managing mediastinal tumors. There are several types of mediastinal tumors, and a single high-magnification histopathological image may contain millions of cellular features. AI can process a large amount of complex information in a short time with the available data entered, and efficiently analyze the tissues with complex lesions or atypical tissues.

Mediastinal sampling can also be easier using AI. The sampling can be achieved by endoscopic techniques, including endobronchial ultrasound-guided transbronchial needle aspiration (EBUS-TBNA) and/or endoscopic ultrasound-guided fine needle aspiration (EUS-FNA) for further histopathological analysis [42]. A presently available real-time endoscopic diagnostic support system based on deep learning could enhance endoscopic technology and achieve efficient tissue observation, sampling, and artificial intelligence [43]. Mediastinal tissue sampling is difficult because of sternal obstruction. A non-ideal sample results in misdiagnosis. Therefore, other mediastinal sampling methods using AI are currently being explored. In a prospective study by Kumar et al. [44], a biopsy needle was inserted into the target lesion with the assistance of an automated robotic arm, and tissue sampling was performed at the site of the highest metabolic activity after confirming the needle’s position by PET/CT imaging.

AI could also be applied in the emerging field of molecular diagnostic pathology. Differential diagnosis of some mediastinal malignancies is very complicated. For example, mediastinal sarcomas are difficult to diagnose because these tumors are relatively rare and possess overlapping clinical and histological features. Several previous studies found that different subtypes had diverse incidence rates, rendering the diagnosis more difficult [45,46,47,48]. Many of these tumors had unique, recurring genetic abnormalities, and cytogenetic and molecular testing assisted in accurate diagnosis [49]. Appropriate AI models could simplify and optimize the identification process. As demonstrated by Capper et al. [50], a specially trained random forest classifier using tumor DNA methylation profile significantly improved the prediction accuracy in hard-to-diagnose subclasses of central nervous system (CNS) cancers (AUC = 0.99).

Generally, the application of AI in managing mediastinal malignancies is very competent, given their associations with complex and recurrent mutations. Critical characteristics that are difficult to identify or quantify by the naked eye can be extracted, and mutations can be predicted from the histopathological images more cost-effectively compared to direct sequencing. Nevertheless, due to limited experience, the existing artificial intelligence may not accurately assess the relatively rare or abnormally classified mediastinal tumors, but it could be considered an auxiliary diagnostic tool. With the advancement of this technology and increasing the number of cases, artificial intelligence will become a powerful application in pathological diagnosis.

## 3. Treatment of Mediastinal Malignancies

### 3.1. Surgical Resection

Surgery is the main treatment option for mediastinal malignancies [51,52]. In traditional open surgery, the integrity of the sternum or chest wall is often altered following surgery and the incision is large, mostly a longitudinal split on the sternum or a large thoracotomy incision to completely expose the area, resulting in a long operation time, postoperative pain, and peri- and postoperative complications [53,54]. Since the 1990s, minimally invasive techniques such as VATS have gained popularity due to their advantages of smaller incisions and quicker recovery times [55]. However, its application has been limited by the distorted two-dimensional field of view and difficult lever-type procedure [56]. Additionally, the mediastinal space is narrow, requiring long thoracoscopic instruments. Hand tremor easily results in errors in judgment, and some areas cannot be reached, making the resection of tumor mass extremely laborious. Da Vinci robot-assisted surgery was invented, improving the accuracy and stability of surgical procedures. It provides a good ergonomic experience and high-definition 3D vision, which is preferred by surgeons. Presently, robotic systems have not yet used machine learning and deep learning, but have laid the foundation for the application of artificial intelligence in surgical procedures.

The current use of AI in handling mediastinal malignant tumors focuses on preoperative planning, covering the feasibility and scope of surgery. Clinical images provide apparent features for the analysis. Subsequently, fast and accurate feature extraction can be achieved through deep learning algorithms, and intelligent segmentation can be completed to promote high-precision identification and accurate matching of anatomical sites, achieving efficient and accurate preoperative planning [57,58,59]. Yohei et al. [60] created an artificial intelligence model using an automatic machine learning platform to predict the mediastinal lymph node metastasis, regulating the surgical procedure, with a prediction accuracy rate of 84%. Although the model had a low sensitivity value of 12%, AI had the ability and potential to complement the shortcomings of existing modalities.

A combination of robotic systems and AI is the future tool in the medical field. The complex structure of the mediastinum makes it easy to injure the spinal cord, cervical nerve roots, hilum of the lung, and unnamed vessels and nerve branches during the surgical approach, and renders fat removal during the surgical procedure difficult [61,62,63]. Completely depending on humans is not reliable enough. Uses of robotics have shown advantages over traditional mediastinal surgery pertinent to precision and stability [64,65]. AI has the potential to assist in determining the best surgical strategy and optimal surgical path [66], which may lead to a reduction in the incidence of complications such as muscle weakness, pneumonia, respiratory failure, and pulmonary edema [67,68,69].

There is still substantial room for improvement in the existing AI for handling mediastinal malignancies. The sensitivity is far from satisfactory. Furthermore, there is still a long way to go toward universal adoption. With the optimization of equipment and the improvement in operation technologies, AI-based robot-assisted surgery may soon become a routine surgical method.

### 3.2. Chemotherapy

The application of artificial intelligence in chemotherapy has many attributes. First, chemotherapeutic drugs can be improved. Some successes in the improvement of chemotherapeutics have been reported in cancer treatment. Berishvili et al., created a deep neural network algorithm to develop anticancer drugs inhibiting PI3Ks and tanker enzymes, promising targets for colorectal cancer (CRC) treatment [70]. Using DNN, Sakellaropoulous et al. [71] trained a model using cancer drug sensitivity genomics (GDSC) cell lines and then used it to predict the responses to paclitaxel in these cells and other mediastinal malignancies. Mediastinal tumors are diverse and suitable for different chemotherapeutic drugs. By improving the efficacies of drugs through artificial intelligence, database-based comparisons and analyses can be efficiently achieved, and the time of conducting research can be reduced. Artificial intelligence can also reduce the cost of research and development and enhance treatment efficacy [72].

AI can optimize individualized chemotherapy regimens to the greatest extent possible. Drug resistance is a serious problem in chemotherapy, resulting from insufficient concentrations of the drugs circulating through the bloodstream to reach the tumor. AI can predict the drug and its response using its learning ability, keeping the drug concentration above a threshold [73]. In 2008, an American biopharmaceutical company developed an AI platform to identify the potential drug targets in cancer patients and maximize the efficacies of cancer drugs [74,75]. A previous study successfully determined the optimal doses of ZEN-3694 and enzalutamide using “CURATE.AT”, an artificial intelligence platform created by the National University of Singapore using technologies, such as deep learning, to improve the efficacy and tolerability of combination therapies [76]. When chemotherapeutic drugs and nanorobots are functionally combined, nanoparticles act as drug carriers to accurately target the cancer cells to specifically bind, reducing the drug dosage and decreasing the side effects of chemotherapeutics [77]. Overall, artificial intelligence can simplify complex mediastinal tumor chemotherapy with the assistance of built robots, AI platforms, and other ways to develop the best-personalized management plan.

### 3.3. Radiotherapy

Many mediastinal tumors, such as thymoma, have a high risk of postoperative recurrence [78]. Radiotherapy is effective as an adjuvant treatment but poses many challenges, including a shortage of radiologists and patients’ intolerance to radiotherapy. AI could solve these problems in various ways.

The first is the determination of organs at risk (OARs) and the tumor size [79]. The goal of radiotherapy is to increase the benefit, but with a minimum possible radiation dose to non-diseased areas. Mediastinum is a narrow space, consisting of complex structures surrounding vital organs. With the insufficient target dose and deviated target structure, serious consequences, such as poor treatment effects and side effects, may emerge. Untoward consequences rely on the experience of the radiation oncologist, and precision radiation to the target mass is time-consuming and laborious. AI-based radiomics can achieve automatic delineation of mediastinal tumors, minimizing damage to the surrounding vital organ structures. Previous studies found that the average Dyce similarity coefficient (DSC) for manual delineation was 0.78–0.93, while that for automatic delineation was 0.97–0.99. The degree of variation of manual delineation was relatively high [80]. In a study by Lustberg et al. [81] with automatic segmentation of mediastinal tumors based on the atlas and deep learning, the DL took half of the time (10 min) compared with the manual delineation. AI had obvious advantages over manual target area delineation.

**Figure 2:**
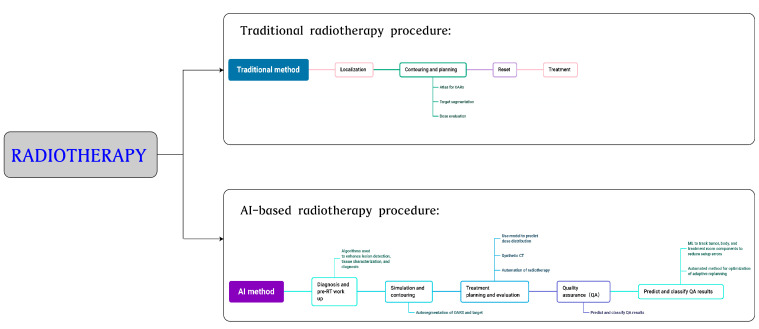
Comparison of radiotherapy procedures between traditional and AI-based radiation methods.

Second, AI can formulate radiotherapy plans. DL-based algorithms predict personalized 3D doses, which can be used to determine treatment dosages and precise locations of mediastinal malignant tumors and other tumors [82,83,84]. Individual bionic characteristics can be used in AI as a basis for making radiotherapy decisions. AI can also be used for assessing and adjusting radiotherapy. Precision radiotherapy, including adaptive radiotherapy (ART), has rapidly developed in recent years. Clinical images can be monitored, the radiotherapy method and prescribed dosage can be adjusted based on tissue changes, and radiotherapy planning can be optimized [85]. Schwaab et al. [86] integrated motion tracking based on ultrasound into heavy ion radiotherapy, used artificial neural networks to estimate the dosage distribution pixel by pixel, and compensated for the delay of about 200 ms between the target motion and position data, so that ultrasound could track the tumor location in real time, guaranteeing the accurate implementation of radiotherapy. Other applications of AI in radiotherapy include predicting radiation-induced toxicity, reconstructing images, and registering images [87,88,89,90]. Although AI is widely used in radiotherapy, it is focused on head and neck cancers and prostate cancers, but studies on mediastinal tumors are limited. Many studies are still in the theoretical stage with a lack of clinical knowledge. The use of AI can effectively increase treatment accuracy and efficiency, reduce complications, and accommodate the automation and intelligence of radiotherapeutic processes in the near future (Figure 2).

## 4. Prognostic Analysis

Prognosis is the prediction and estimation of the consequences associated with the future recurrence of the disease [91]. Oncologists need to predict survival rate, survival time, and other consequences based on clinical evaluation. This prediction carries large contingency and uncertainty. The capability of the doctor, the cognitive level of the patient, the decision related to treatment, the patient’s age, and general health and psychological status are bound to the prognostic decision. Many force majeure events also increase the forecasting difficulty. With the help of AI, prognostic prediction accuracy can be improved. The DL algorithm that is used to automatically extract the features from medical data to build models may accurately predict the risk of tumor recurrence and patient response to treatment [92,93]. Many previous studies have achieved remarkable results in predicting tumor prognosis. The support vector machine system can be used to estimate the five-year survival rate of cancer patients. Zhong et al. [94] carried out a prognostic assessment of lung cancer-specific survival and the possibility of mediastinal lymph node metastasis using deep learning survival neural network models. Previous studies have also found that the prediction accuracy of AI was higher than that of linear regression analysis. In reference to Matsuo et al. [95], they utilized tumor characteristics and laboratory test data from 768 cancer patients to predict both progression-free survival (PFS) and overall survival (OS). The results indicated that the AI model had superior predictive ability compared to the Cox proportional hazards regression model. Sailer et al. [96] compared 10 common data mining algorithms to predict a binary target for five-year survival based on seven attributes, including sex and IACC stage. The average accuracy of ML was 67.7%, which was slightly higher than that of the clinician’s assessment of 59%.

Various studies have demonstrated the superiority of AI in prognostic analysis. Prognostic accuracy is not only useful for scientific research, but is also closely related to the survival status of patients. AI was better in predicting poor prognosis in patients with poor cancer-specific survival (adjusted hazard ratio = 3.04) compared to predicting good prognosis [97]. Intelligent health management can also, with the assistance of AI and data integration analysis, propose different intervention plans and follow-ups for medical services for patients, ensuring a smooth treatment process. AI also provided additional avenues for dynamically adjusting drug dosages with single or combination therapies in individual patients using patient-specific data points collected over time [98]. Furthermore, mediastinal malignant tumors are prone to relapse. Considering malignant thymomas as an example, they are difficult to treat, even by surgery, radiotherapy, and other comprehensive treatments, and result in greater odds of recurrence and metastasis. Moreover, emphysema and other complications, such as infection, are further problems. An accurate and efficient prognostic assessment undoubtedly improves the survival rate and prolongs the survival time in mediastinal malignant tumors.

Although AI makes breakthroughs in prognostic prediction, there are also disadvantages. First, although the prognostic accuracy exceeds that of humans in some studies, there is still a substantial need for improvement in achieving error-free results in diagnosis and other aspects. AI-related research is still in its infancy, the dataset is small in scale and single in type, and the knowledge of AI and clinical expertise are relatively limited. Therefore, it is difficult to develop models that fit the clinical situation and AI [99,100,101]. International multicenter studies should be performed in the future to construct robust algorithms and achieve in-depth communication across disciplines to organizationally combine clinical research [102].

## 5. Prospects of Using Artificial Intelligence

Artificial intelligence can be incorporated into all aspects of mediastinal tumor research and clinical management. At present, one of its potential applications in mediastinal malignancies is to design new anticancer therapeutics or to guide the development of therapeutics. Cancer drug development without doubt greatly benefits from the use of AI. Machine learning algorithms can screen relevant data from a large number of patients during treatment and assist to develop new drugs for improving treatment response, as well as drug resistance and drug side effects. Different drug combinations can be evaluated for their efficacies [103,104]. Drug development often requires the collection of several years of data, which is a complicated process. Machine learning, on the other hand, can process the data within a short period, greatly reducing the time to develop drugs [105,106,107,108]. Zhavoronkov et al., developed a DL algorithm model and found a powerful inhibitor of DDR1, a kinase target involved in a variety of cancers, in 21 days, compared with the time of about a year by conventional processes [109]. In addition to the neural network models that focus on drug molecule production, non-neural network models have remarkable power in predicting drug responses [110]. At present, multiple random forest models, support vector machine models, and other algorithm models have been developed with the capability of predicting various characteristics of drugs, including toxicities, adverse reactions, absorption, metabolism, and excretion [111,112]. AI could also enable the reuse of anti-cancer drugs. Antagonistic autoencoders are applied to full-dose response data measured in cell lines to develop deep-learning models. In addition, new methods such as integrated cell signature libraries (LINCS) have been used to develop transcriptional datasets to facilitate the reuse of datasets [113,114]. Some types of neural networks, including autoencoders, can elucidate the sets of molecules that represent certain activities and generate new knots with similar activities. The use of AI to understand the molecular mechanisms of drugs, estimate the effects of drugs, develop new drugs, and reuse drugs is critical to the future treatment of mediastinal tumors.

Furthermore, AI can sequence cancer genes and analyze the relationship between genotypic and phenotypic characteristics, to understand the biological basis of mediastinal tumors. Davis et al., found a role for F-box/WD repeat protein 7 (Fow7) in the oxidative metabolism of cancer cells through the analysis of gene expression characteristics of the cancer Gene Atlas dataset [115]. Gene mutations associated with mediastinal malignant tumors can be detected by using related algorithms. DeepVariant, a DNN-based method, first generates a read stack image for a candidate variant (making an image classification task) and then predicts its genotype status (homozygous reference, heterozygous variant, and homozygous variant) to detect the variants read by NGS [116].

The application of AI in cancer also includes the collection of comprehensive data, which is the basis of machine learning for cancer diagnosis, treatment, and prognostic analysis. Patients’ data, such as demographic information, family history, clinical symptoms, comorbidities, histopathological features, immunohistochemistry results, nucleic acid sequencing, biochemical analysis, digital images, and empirical measurements are collected using digital devices, and statistical and mathematical models are constructed [117]. Based on these data, data integration and analysis are performed to facilitate the tracking of long-term patient information [118,119].

Multi-omics integration will also be a key feature for the combination of mediastinal tumors and AI in the future. Data sources from different omics platforms are normalized, compared, and analyzed to establish the relationships among various groups, and a comprehensive and in-depth interpretation of biological processes at the gene, transcriptional, protein, and metabolic levels is carried out by integrating multiple omics data, to better understand the biological systems [120,121,122].

In conclusion, many pan-cancer events and machine learning combinations are currently being attempted, suggesting the infinite possibilities of application in handling mediastinal malignant tumors. However, mediastinal cancer is complex from its definition to its specific treatment. It is significant to use various learning methods and different data granularities to identify the right way to manage mediastinal malignancies in clinical practice. Breakthroughs can be made with the help of artificial intelligence in the diagnosis, treatment, and prognostic evaluation of mediastinal cancers [123,124].

## 6. Summary

Although AI has made rapid progress in recent years, its application in oncology is still full of unknowns and challenges. The application of AI in different cancers is disproportionate, and enough attention has not been paid to mediastinal tumors, showing a huge gap and potential for future development. The use of artificial intelligence in various fields, including the medical field, still presents many challenges, such as the lack of standardization, the immaturity of the technology, the high cost, the controversies in moral and ethical aspects, and poor supervision. It should be clear that AI could not become a remedy for all mediastinal tumors, or completely replace the analysis with human intelligence. However, AI can be gradually applied in the diagnosis, treatment, and prognosis assessment of mediastinal tumors to produce remarkable improvements. We firmly believe that there will be more AI-involved tasks in the medical field in the future, with generalization, accuracy, and stability, benefitting humans.

## Data Availability

All the materials and information will be available upon request by e-mail to the corresponding author.

## Supplementary Materials

The following supporting information can be downloaded at: https://www.mdpi.com/article/10.3390/jcm12082818/s1, Table S1: Application of artificial intelligence in diagnosing mediastinal malignant tumors.
