# Supplementary material for: Application of Artificial Intelligence in the Diagnosis, Treatment, and Prognostic Evaluation of Mediastinal Malignant Tumors
Source: J Clin Med. 2023 Apr 11;12(8):2818. doi: 10.3390/jcm12082818 (PMC10144939; doi:10.3390/jcm12082818)
Supplement: Supplementary file 1 [file jcm-12-02818-s001.zip › jcm-2143551-supplementary.pdf]

Table S1. Application of artificial intelligence in diagnosing mediastinal malignant tumors

Authors	Year	Country/Region	Number of patients in the study	Tumor types	Results	References
Ozkan et al.	2022	Turkey	27	Thymomas	AUC (0.83)	[20]
Dai et al.	2020	China	137	Thymic tumor	Accuracy (94.73%)	[21]
Lin et al.	2022	Taiwan, China	62	Thymic tumor	Sensitivity: lymphoma (52.9%), thymoma (74.2%), and thymic carcinoma (92.8%)	[22]
Chowdhary et al.	2017	India	64	Thymomas	Overall Accuracy (87%) (AUC = 0.90)	[23]
Kalra et al.	2020	Canada	124	Thymoma	Accuracy (100%)	[29]
